# Heterogeneous reactions in a HFCVD reactor: simulation using a 2D model

**DOI:** 10.3762/bjnano.15.128

**Published:** 2024-12-17

**Authors:** Xochitl Aleyda Morán Martínez, José Alberto Luna López, Zaira Jocelyn Hernández Simón, Gabriel Omar Mendoza Conde, José Álvaro David Hernández de Luz, Godofredo García Salgado

**Affiliations:** 1 CONAHCYT-Posdoctorado-Centro de Investigaciones en Dispositivos Semiconductores (CIDS-ICUAP), Benemérita Universidad Autónoma de Puebla (BUAP). Col. San Manuel, Cd. Universitaria, Av. San Claudio y 14 sur, Edif. IC5 y IC6. Puebla, Pue., 72507 México; 2 Centro de Investigaciones en Dispositivos Semiconductores (CIDS-ICUAP), Benemérita Universidad Autónoma de Puebla (BUAP). Col. San Manuel, Cd. Universitaria, Av. San Claudio y 14 sur, Edif. IC5 y IC6. Puebla, Pue., 72507 México

**Keywords:** 2D model, chemical reactions, flow dynamics, HFCVD, hot filament chemical vapor deposition, SiO*_x_* films

## Abstract

In this study, a simulation of the elementary chemical reactions during SiO*_x_* film growth in a hot filament chemical vapor deposition (HFCVD) reactor was carried out using a 2D model. For the 2D simulation, the continuity, momentum, heat, and diffusion equations were solved numerically by the software COMSOL Multiphysics based on the finite element method. The model allowed for the simulation of the key parameters of the HFCVD reactor. Also, a thermochemical study of the heterogeneous reaction between the precursors quartz and hydrogen was carried out. The obtained equilibrium constants (*K*_eq_) were related to the temperature profile in the deposition zone and used in the proposed simulation. The validation of the model was carried out by measuring the temperature experimentally, where the temperature range on the substrate is 450 to 500 °C for different deposition parameters. In the simulation, the laminar flow of species contributing to the film growth was confirmed, and the simulated concentration profiles of H° and SiO near the filaments and the sources were as expected. H° and SiO are essential species for the subsequent growth of the SiO*_x_* films. These SiO*_x_* films have interesting properties and embedded nanostructures, which make them excellent dielectric, optoelectronic, and electroacoustic materials for the fabrication of devices compatible with silicon-based technology.

## Introduction

The growth of materials such as non-stoichiometric silicon oxide (SiO*_x_*) is an important step in semiconductor devices development. Control of deposition parameters determines the success of the process and the properties of the films, with the most important parameters being substrate temperature, gas pressure, species concentration, and flow velocity [1]. The structural, optical, and electrical properties of the SiO*_x_*, more generally known as silicon-rich oxide (SRO), films are determined by the ratio *x* = [O]/[Si], which is determined by controlling key parameters in the deposition process [2]. This ratio determines optical and electrical properties such as bandgap energy, absorption coefficient, photoluminescence, refractive index, and electrical conductivity [3]. SiO*_x_* cannot only be obtained by different CVD techniques, but also by sputtering and ion implantation, among others [4–5]. The key parameters are particular for each of these techniques. Hot filament chemical vapor deposition (HFCVD) is an excellent alternative for obtaining SRO films. It is also very versatile and economical because the input gases and materials are accessible; also, it is scalable to larger areas [6].

The SiO*_x_* films obtained by HFCVD possess excellent optical and electrical properties, which makes such films suitable for applications in the manufacture of metal–insulator–semiconductor and metal–insulator–metal devices exhibiting blue and white electroluminescence [7–8]. It was found that these films exhibit photoconductive and photoelectric effects suitable for electroluminescence and photovoltaics applications [9–10], as well as for other applications such as solar cells and anodes for Li batteries [11]. The basic steps of the general CVD process are classified and described in [12].

The optimization of this technique improves the properties of the films; however, the complexity of the CVD processes makes it difficult to understand clearly the different mechanisms involved in such optimization. Different tools such as ab initio density functional theory [13–14], kinetic Monte Carlo simulations [15–16], and reactive molecular dynamics simulations [17–18] have been used to understand the chemical reactions underlying the growth of the films [19].

Modeling the reaction mechanism in both two-dimensional (2D) and three-dimensional (3D) systems is a tool that allows us to understand the key steps regarding the reproducibility and uniformity of the films [19–20]. From a computational point of view, prior works focused on the growth mechanism of SiO*_x_* in a plasma-enhanced chemical vapor deposition reactor in zero dimensions (0D); the model used was solved using CHEMKIN III and AURORA software. In the model, a set of reactions was established that approximately describe the mechanisms of the material growth, and the model results were compared with those obtained by experimental measurements [21]. Also, modeling of CVD microreactors at atmospheric pressure using tetraethyl orthosilicate as a source to obtain SiO_2_ has been achieved through computational fluid dynamics (CFD) simulations [22]. The gas-phase and surface reactions were analyzed using direct Monte Carlo simulations of a hot wire chemical vapor deposition reactor for the growth of polycrystalline SiO_2_ [23]. Most of these models describe CVD reactors at low pressure and low temperature, but there are not enough models regarding CVD systems at high temperature (>800 K) and high pressure (atmospheric pressure).

In this investigation, we focus on the simulation and analysis of key steps in a HFCVD deposition process to obtain SiO*_x_* films by means of continuity, momentum, heat, and diffusion equations, which were solved numerically by the software COMSOL Multiphysics based on the finite element method; we also carry out a thermochemical analysis using FactSage. Some of the theoretical results are compared to experimental results. This work consists of five sections apart from this one. In Section “Experimental”, there is a complete description of the HFCVD reactor and the deposition parameters. Section “Theoretical and Numerical Simulations” explains the equations for the 0D and 2D models. Also, the hypothesis used to develop this study and the methodology for the use of COMSOL and FactSage are given. In section “Results and Discussion”, some theoretical results are compared with experimental ones. Further, the results obtained from the simulation are discussed regarding the profiles of temperature, gas velocity, and concentration of the species. Finally, the main conclusions of this research are expressed in section “Conclusion”.

The study focuses on the convective transfer of the reactive gases to the solid source and the surface diffusion to the substrate. The main objective is to optimize the process for an HFCVD reactor and, thus, improve the quality and reproducibility of the films.

## Experimental

The analyzed HFCVD system is a vertical reactor that can be divided into three zones. The first zone is the gas inlet, the second one is the reaction zone, and the third one is the gas outlet. In the first zone, molecular hydrogen (H_2_) gas is pumped in through a stainless steel piping system that reaches a diffuser inside the reaction chamber. The gas gets in contact with eleven tungsten filaments from incandescent lamps, which are activated by an externally applied voltage generating a current; these filaments have a temperature of approximately 2300 K. The second zone includes the region where the chemical reactions takes place. Here, molecular hydrogen, exposed to the high temperature of the filaments, dissociates to form atomic hydrogen, which reacts with the eleven solid quartz sources. A cloud or plasma is formed and finally reaches the substrate for the formation of the thin films. Finally, zone three is the exit of the gases that were not deposited in the film. The entire process is carried out under atmospheric pressure. Table 1 summarizes the values of the parameters and dimensions complementary to the experimental conditions in the reactor for the deposition of SRO films previously described and depicted below in Figure 2 [24], as well as the corresponding boundary conditions.

**Table 1:** Elements, dimensions, and parameters of the deposition in the HFCVD reactor.

Parameters and dimensions of the HFCVD reactor		Deposition parameters and boundary conditions	
	
parameter	value	parameter	value
	
filament diameter	0.5 [mm]	temperature of the filament (no-slip wall)	2000 [°C]
filament length	10 [mm]	pressure (outlet)	1 [atm]
distance between filaments	3.8 [mm]	mass flow (inlet)	25, 50, and 100 [sccm]
deposition time	3 [min]	substrate temperature (no-slip wall)	26 [°C]
reactor volume	0.009 [m^3^]	distance source–substrate (DSS)	3, 4, and 5 [mm]
surface area of the sources	2.19 × 10^−5^ [m^2^]	distance filament–source	6 mm
		substrate diameter	50.8 mm

## Theoretical and Numerical Simulations

### Hypothesis

Obtaining non-stoichiometric silicon oxide films in a HFCVD reactor is mainly based on two main heterogeneous reactions, which are the dissociation of atomic hydrogen and the reaction of atomic hydrogen with a solid source. The thermochemical study will allow us to obtain thermodynamic parameters of the heterogeneous reaction between H° in the gas phase and quartz to describe its behavior as a function of temperature at constant pressure. The thermodynamic data obtained and those taken from the bibliography will be used in the Arrhenius equation to calculate the thermodynamic and transport properties of the system in the gas phase in a dimensionless and temporal model. The thermodynamic and transport properties of the mixture will permit a study in the steady state considering the dimensions of the HFCVD reactor and the deposition parameters through numerically solving the equations of continuity, momentum, and heat transfer by the finite element method.

### Mathematical method and equations

**Figure 1:**
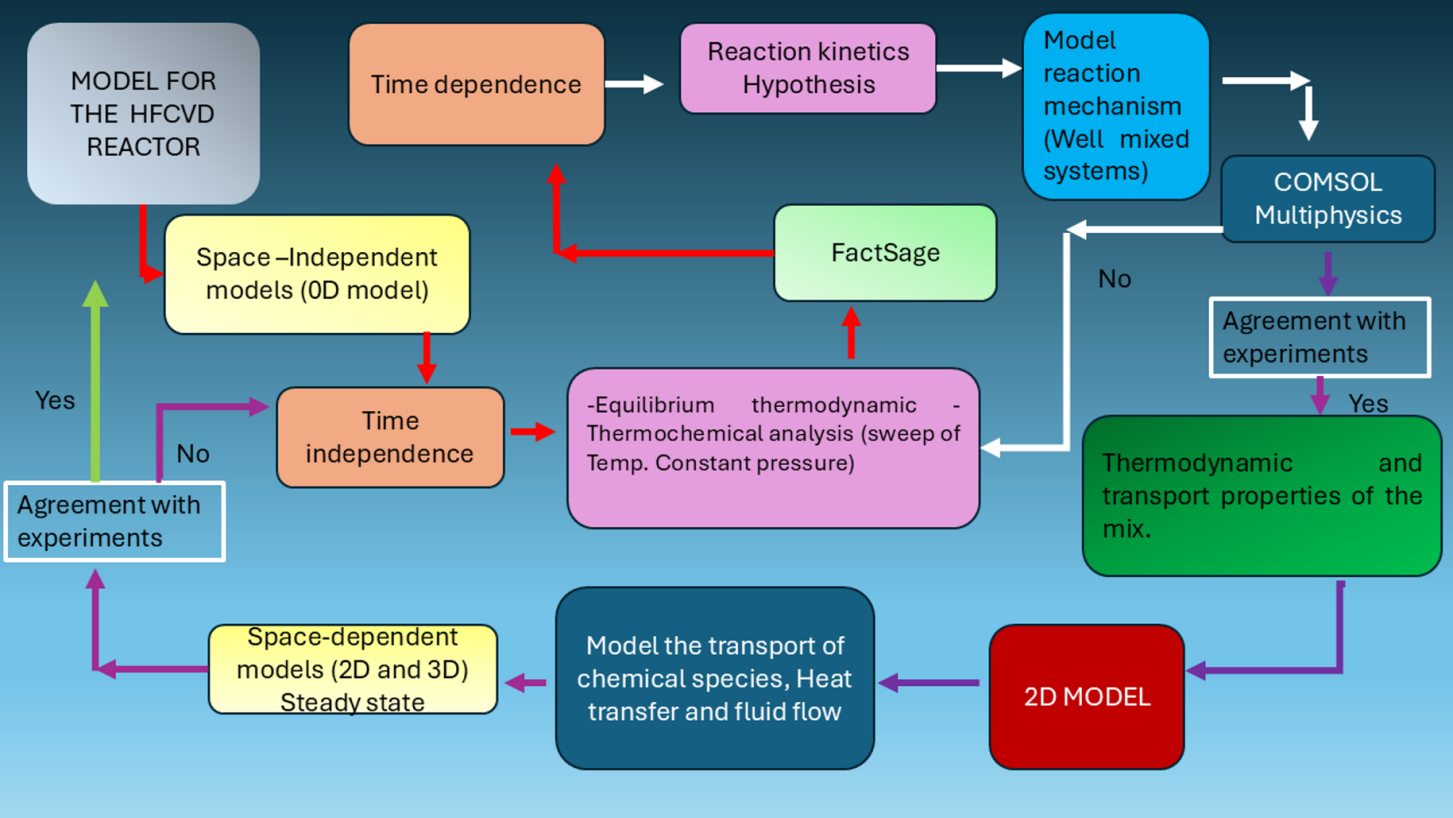
Flowchart of the modeling and simulation process and the use of different pieces of software.

The complex growth of non-stoichiometric silicon oxide films in a HFCVD reactor involves different physics. For the description of the behavior of all systems, it is necessary to incorporate mathematical models that can explain the different phenomena involved. In this section, the mathematical models and the different pieces of software used in this study will be described. Figure 1 shows a flowchart that describes the modeling and simulation process. The different mathematical models used, and their mathematical equations will be described in detail in the following sections.

#### The well-mixed reactor equations (0D model)

The modeling of reactions is based on the mass action law given by Equation 1:


[1]

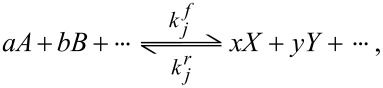



where *A*, *B*,… are the reactants and *X*, *Y*,… are the products. *a*, *b*,… are the stoichiometric coefficients of the reactants, and *x*, *y*,… are the coefficients of the products. In the case of a set of reactions, the reaction rates *r_j_* (mol·m^−3^·s^−1^) can be described by the law of mass action given by Equation 2:


[2] $$r_{j}=k_{j}^{f}\prod_{i\in \text{react}}c_{i}^{-v_{ij}}-k_{j}^{r}\prod_{i\in \text{prod}}c_{i}^{v_{ij}},$$

where 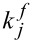 and 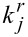 refer to the forward and reverse rate constants, respectively. The concentration of species *i* is indicated as *c_i_* (mol·m^−3^). The stoichiometric coefficients are expressed by *v_ij_* and are negative for reactants and positive for products [25].

The dependence of concentration and temperature on reaction rates can be included using the Arrhenius expression (Equation 3):


[3] $$k=AT^{n}e^{\left(-E_{\text{A}}/RT\right)},$$

where *A* is the frequency factor, *T* (K) is the absolute temperature, *n* is the temperature exponent, *E*_A_ (J·mol^−1^) is the activation energy, and *R* is the gas constant (8.314 J·mol^−1^·K^−1^).

For chemical reactions in equilibrium, the equilibrium constants are defined in terms of the equilibrium expression *K*_eq_ by Equation 4:


[4] $$K_{\text{eq}}=\prod_{i}c_{i}^{v_{i}}=\frac{\prod_{i\in \text{prod}}c_{i}^{v_{i}}}{\prod_{i\in \text{react}}c_{i}^{-v_{i}}}.$$

The constants *K*_eq_ were calculated through data obtained below from Table 2 and using Equation 5:


[5] $$\Delta G{}^{\circ}=-RT\ln K_{\text{eq}},$$

where Δ*G* is the change in the Gibbs energy and *R* is the gas constant.

#### The equations in the spatial model

The used model assumes conservative species transport by diffusion and convection through a mass balance as described by Equation 6:


[6] $$\nabla \cdot J_{i}+u\cdot \nabla c_{i}=R_{i}.$$

In this equation, **J***_i_* (mol·m^−2^·s^−1^) is the diffusive flow vector, *R_i_* (mol·m^−3^·s^−1^) is a rate expression for the species, and **u** (m·s^−1^) is the mass-averaged velocity vector. The suffix *i* denotes the species.

The laminar behavior of the gas-phase fluid is based on the Navier–Stokes equations; for an incompressible flow, ρ = constant. The continuity equation in the general form is expressed by Equation 7, the momentum equation in the general form is given by Equation 8, and the transfer of heat in fluids is described by Equation 9:


[7] ∇⋅(ρu)=0,

[8]

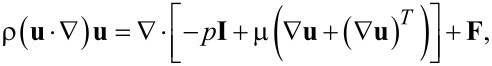




[9]

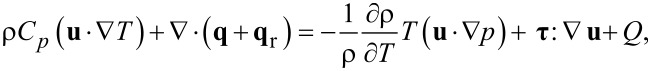




[10] $$\tau :\nabla u={\sum_{i}\sum_{j}\left[\frac{\tau_{ij}}{2}+\frac{1}{3}\nabla \cdot u\delta_{ij}\right]}^{2},$$

[11] $$\tau_{ij}=-\mu \left(\frac{\partial u_{i}}{\partial x_{j}}+\frac{\partial u_{j}}{\partial x_{i}}\right),$$

where **u** is the velocity vector (m·s^−1^), ρ is the density (kg·m^−3^), *p* is the pressure (Pa), **F** is the volume force vector (N·m^−3^), *C_p_* is the specific heat capacity at constant pressure (J·kg^−1^·K^−1^), *T* is the absolute temperature (K), **q** is the heat flux vector (W·m^−2^), **q**_r_ is the heat flux vector by radiation (W·m^−2^), **I** is the identity matrix (unitless), (∇**u**)*^T^* is the transposed velocity gradient tensor, **τ** is the viscous stress tensor (Pa), μ is the dynamic viscosity (Pa·s), *Q* includes heat sources other than viscous dissipation (W·m^−3^), and δ*_ij_* is the Kronecker delta symbol. All equations in this section were taken from [26], except Equation 5, which was taken from [27].

### The modeling of chemical reactions

A prior thermodynamic equilibrium study of the heterogeneous reaction of SiO_2_ (s) + H° (g) at 1 atm pressure in the temperature range from 100 to 2000 °C using FactSage software found that the gas species with higher concentrations were H°, SiO, OH, H_2_O, and O_2_; those with lower concentrations were SiH_4_, SiH, Si, O, Si_2_, and Si_3_ [24]. This study was the basis for establishing the four main chemical reactions in zone 2 in the HFCVD reactor, which are listed below in Table 3. Here, an additional thermochemical study of the reaction SiO_2_ (s) + H° (g) was developed to obtain the extensive properties of this reaction using FactSage in the temperature range from 500 to 1500 °C. Enthalpy (*H*), Gibbs energy (*G*), entropy (*S)*, heat capacity (*C*_p_), and Helmholtz energy (*A*) were obtained to calculate *K*_eq_ through Equation 5 [27]. The thermodynamic properties are listed in Table 2.

**Table 2:** Thermodynamic properties for reaction 3 in Table 3.

Temperature *T* (°C)	Enthalpy *H* (J)	Gibbs energy *G* (J)	Entropy *S* (J/K)	Volume *V* (L)	Heat capacity *C*_p_ (J·K^−1^)	Helmholtz energy *A* (J)	Equilibrium constant *K*_eq_

500	−677803.9	−774752.6	193.897	4.11 × 10^1^	80.284	−778912.2	8.62784 × 10^80^
600	−669523.0	−794911.9	208.981	4.93 × 10^1^	85.279	−799902.9	1.59062 × 10^69^
700	−660742.4	−816497.1	222.507	5.75 × 10^1^	90.434	−822319.6	8.43911 × 10^60^
800	−651404.4	−839377.8	234.967	6.57 × 10^1^	96.539	−846031.8	6.37426 × 10^54^
900	−645606.2	−863342.5	241.929	7.39 × 10^1^	20.828	−870827.9	1.27612 × 10^50^
1000	−643523.4	−887647.1	244.124	8.21 × 10^1^	20.828	−895964.0	2.31640 × 10^46^
1100	−641440.6	−912160.3	246.109	9.03 × 10^1^	20.828	−921308.6	2.05986 × 10^43^
1200	−639357.8	−936863.1	247.921	9.85 × 10^1^	20.828	−946842.9	6.02018 × 10^40^
1300	−637275.1	−961739.7	249.588	1.07 × 10^1^	20.828	−972551.0	4.38780 × 10^38^
1400	−635192.3	−986776.6	251.132	1.15 × 10^1^	20.828	−998419.4	6.54940 × 10^36^
1500	−633109.5	−1011962.0	252.569	1.2311 × 10^1^	20.828	−1024436.7	1.73301 × 10^35^

It should be noted that the surface film growth reactions are not considered in this study. The approximation of the thermodynamic properties of the superficial species is a study that we are still developing.

### Numerical simulation

**Figure 2:**
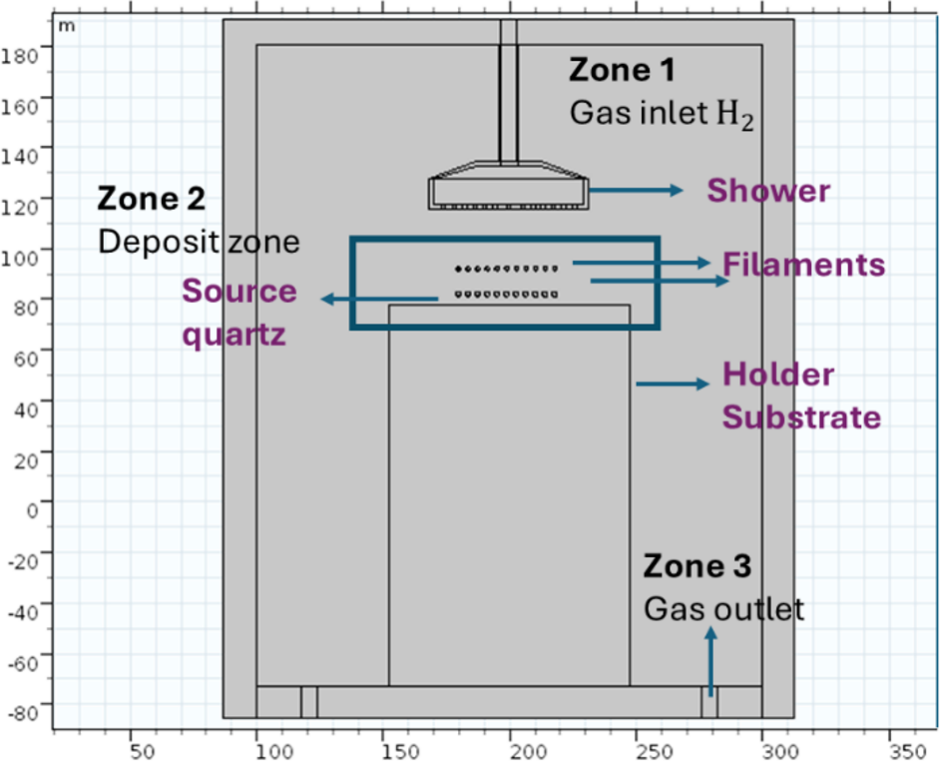
The HFCVD reactor zones and elements of the 2D model built in COMSOL Multiphysics 5.3.

A 2D model of the HFCVD reactor was created using COMSOL Multiphysics 5.3 software [28]. Figure 2 shows the 2D geometry model with the three zones of the HFCVD reactor. For the numerical solution, the finite element method discretizes the domain into a mesh of smaller parts called elements. The computational time for the numerical solution is related to the mesh in the 2D model with longer computational time for a greater number of elements.

Based on the thermodynamic equilibrium study, we propose four main reactions in zone 2, which are listed in Table 3. For the generation of atomic hydrogen, reactions 1 and 2 are considered, where M is a tertiary species or H_2_. Atomic hydrogen diffuses and reacts superficially with the quartz sources; this corresponds to the process described by the heterogeneous reaction 3. Finally, reaction 4 is considered for the generation of OH, considering that the source of oxygen is the interaction of atomic H° with the sources. The Arrhenius parameters used are described in Table 3.

**Table 3:** Reactions for the 0D and 2D models.

Reaction	Frequency factor *A*	Temperature exponent *n*	Activation energy *E*_A_	Ref.

1. H + H + M = H_2_ + M	1.00 × 10^18^	−1.00	0.0	[16]
2. H + H + H_2_ = H_2_ + H_2_	9.2 × 10^16^	−0.6	0.0	[16]
3. SiO_2_ (s) + H° (g) = SiO + OH	4.0 × 10^12^	0.0	5700	[17–18]
4. H_2_ + O_2_ = OH + OH	1.7 × 10^13^	0	48100	[16]

Initially, a well-mixed reactor approach will be used. This is a 0D model in which the reactor volume is assumed to be constant and spatially uniform; the calculated concentrations of the species, and the temperature are instantaneous values within the reactor. The 0D model scales to a spatial 2D model. For the numerical solution, the following considerations are made: Molecular hydrogen behaves like an ideal gas in a two-dimensional model with position coordinates (*x*,*y*). The fluid is considered laminar and incompressible. The mathematical equations that describe the motion of gas are the conservation of mass (continuity equation), momentum (Navier–Stokes equations), and energy. The boundary conditions used in this model are listed in Table 1. Through the finite element method, all equations were solved via COMSOL Multiphysics. The Dirichlet boundary conditions were employed to solve this model numerically. The types of boundaries are listed in Table 1. For the boundary wall on the filaments, we use a constraints boundary condition; for the outlet, a pressure boundary condition was used. For the inlet, we use a mass flow boundary condition.

The simulations give us a broad overview on the probable reaction mechanisms during the deposition of thin films in the HFCVD reactor. This allows us to get an estimate of the types of species that will be deposited for the growth of thin films, which depend strongly on the parameters and precursors employed.

## Results and Discussion

### Experimental results

The SiO*_x_* films obtained using the HFCVD reactor have already been characterized using different optical, electrical, and structural characterization techniques [29–31]. Also, the process of the growth of the films was optimized through a computational fluid analysis study, which made it possible to increase the deposition area, in addition to enhancing the optoelectronic properties of the films [24].

### Theoretical results

**Figure 3:**
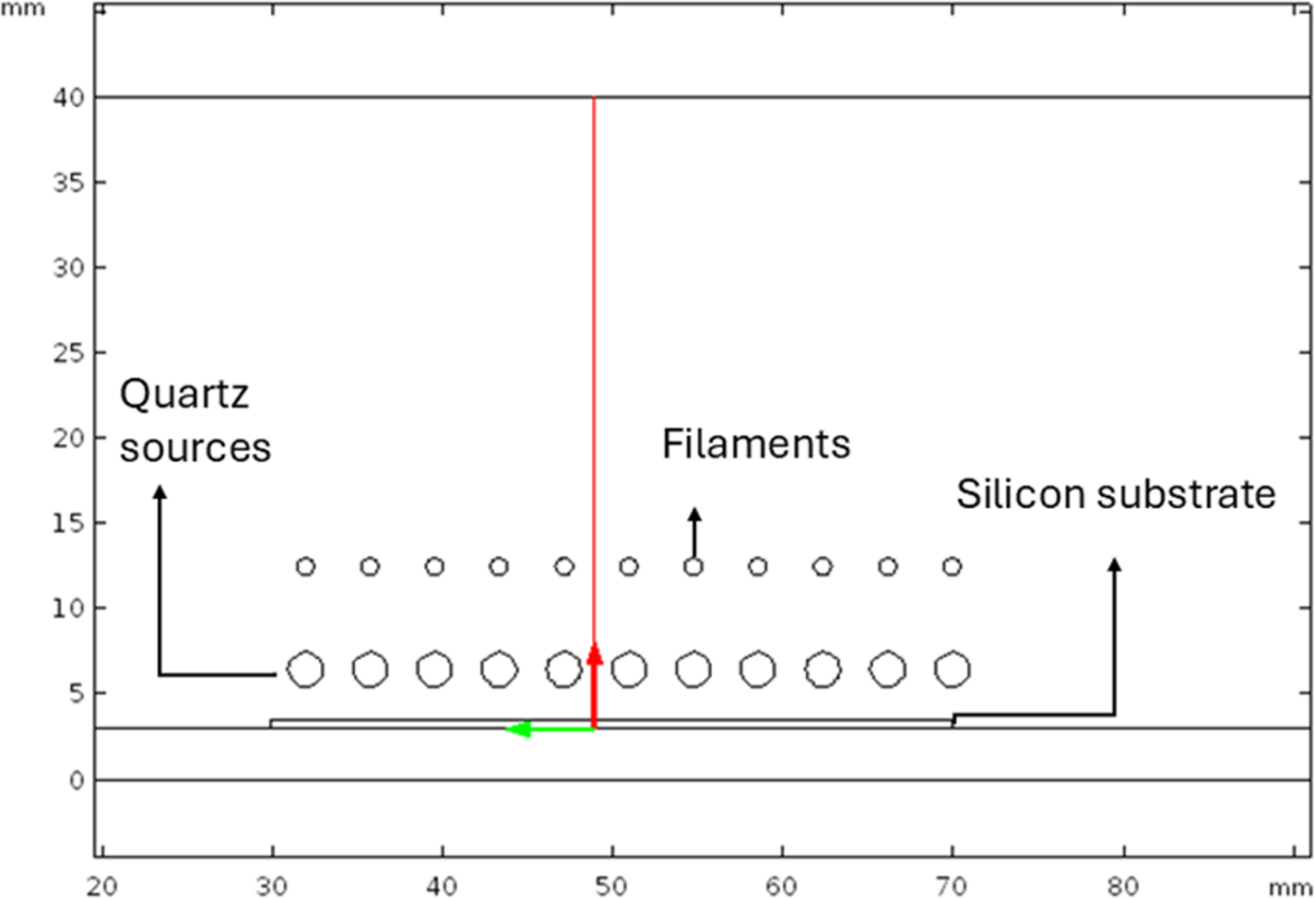
The red line along the *y* direction in the deposition area (zone 2) built in COSMOL Multiphysics.

The 2D model was numerically solved in the steady state with a computing time of 286 s. For the simulation, the 2D model was analyzed with a mesh of 45,769 elements. First, the distribution of temperature and velocity of the fluid and, second, the approximation of the concentration and distribution of the species are presented. All results reported in this research were obtained along a line on the *y* axis of the 2D model in zone 2. The line crosses all horizontal zones of the HFCVD reactor. Figure 3 shows the line in red. The data obtained on the mentioned line is presented and analyzed separately according to the main variables in the HFCVD system during deposition.

### Temperature

**Figure 4:**
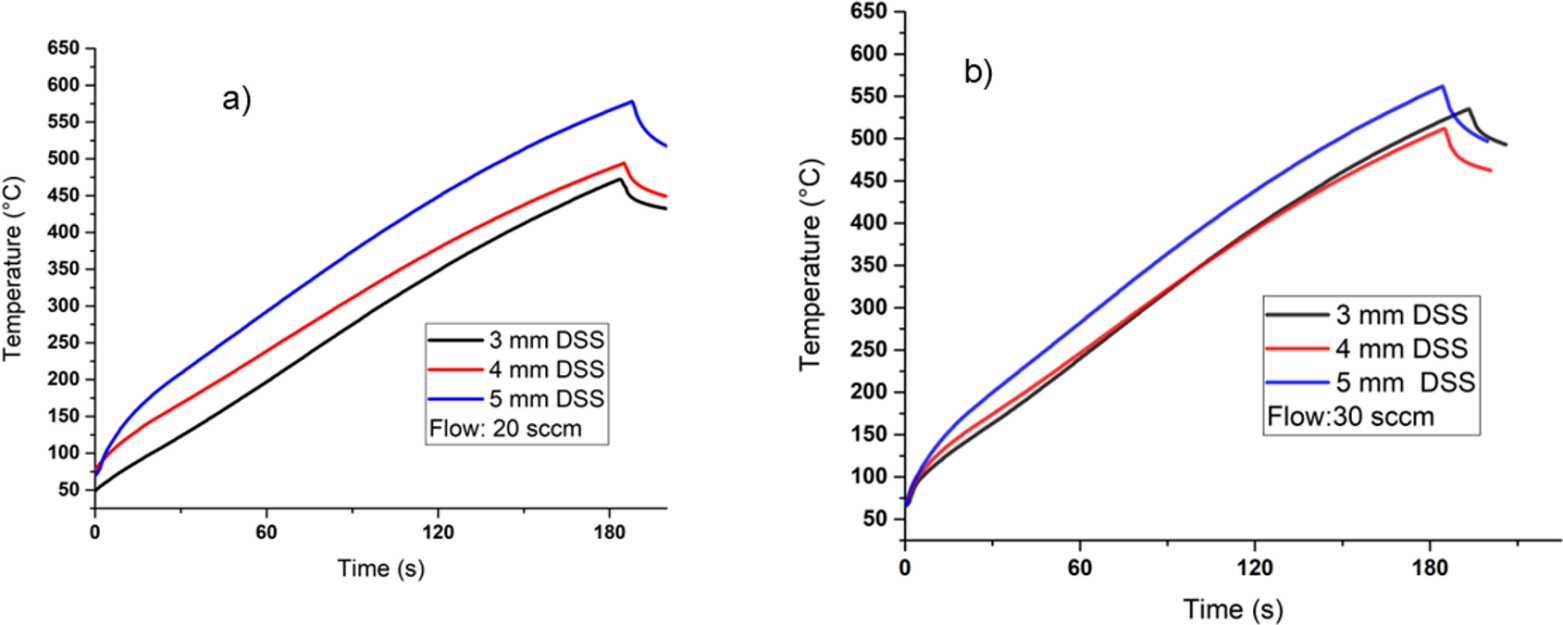
Experimental temperature measured by a thermocouple located on the deposition zone for (a) 20 sccm and (b) 30 sccm.

The theoretical model was experimentally validated through temperature profiles for 20 and 30 sccm flows, which are shown in Figure 4a and Figure 4b, respectively [32]. The temperature measurements were made using a K-type thermocouple located on the surface of the deposition area. The deposition time for SiO*_x_* was 3 min at a fixed position of the thermocouple on the substrate. Thereafter, the position of the sources was moved by 3, 4, and 5 mm. The experimental temperature measurements range from 450 to 550 °C after a deposition time of 3 min considering the mentioned DSSs.

**Figure 5:**
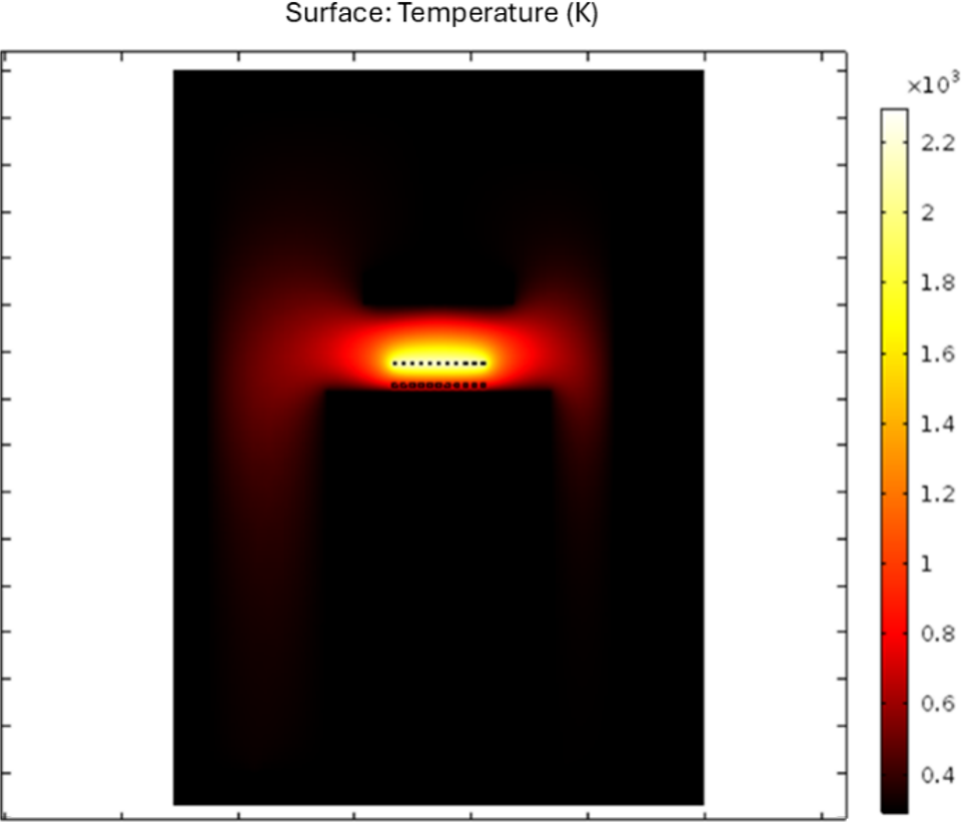
Temperature map of the HFCVD reactor.

**Figure 6:**
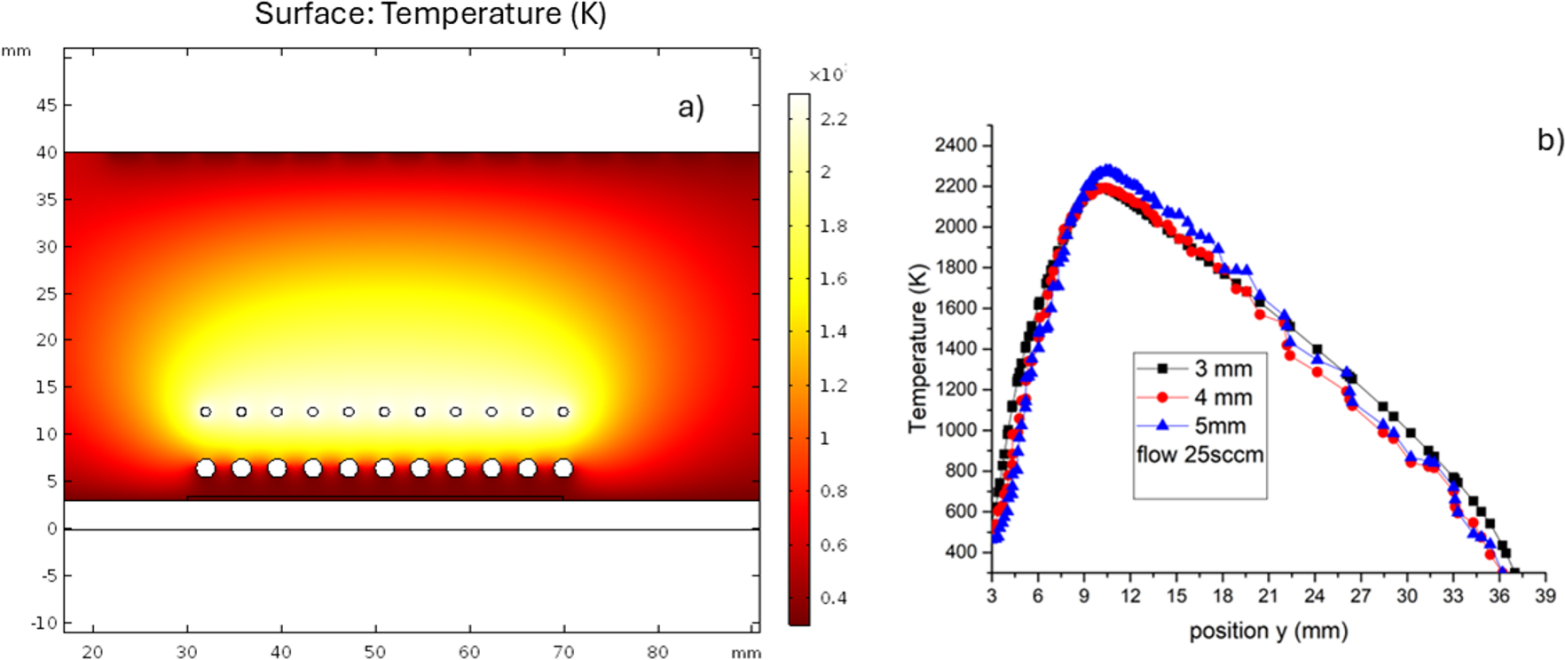
(a) Temperature map of the surroundings of the deposition zone. (b) Temperature profile along the *y* axis from the substrate to the diffuser for the different DSSs in the steady state.

The 2D temperature map of the HFCVD reactor is shown in Figure 5. The temperature map of deposition zone 2 is shown in Figure 6a. Figure 6b describes the temperature profile along the *y* coordinate from the substrate to the diffuser for the different DSSs in the steady state. The maximum temperature is near the filaments, and the minimum temperatures are found on substrate and diffuser. Heat transfer in the reactor is due to different mechanisms, such as radiation, conduction, convection, and diffusion. The theoretical and experimental temperature results were compared and analyzed near the position of the thermocouple, where the experimental temperature is only a function of the deposition time but not of the position. From the theoretical results, the temperature increases from 300 K at the substrate to 1000 K near the quartz sources, that is 3–5 mm away from the sources in the steady state.

The results obtained show us that the HFCVD is a high-temperature system regarding the filaments (2300 K), compared with other CVD reactors like low-pressure CVD (800 K). Hence, diffusion is expected to dominate the reactions for the formation of the precursor species. The growth of SiO*_x_* films is controlled by nucleation effects. According to results for high temperatures over 700 K, the supersaturation is high and the nucleation is homogeneous in the process, leading to the precipitation of solid particles on the substrate and powder formation [23]. SiO*_x_* powders are obtained in the HFCVD reactor when the distance between the filaments and the source is less than 6 mm. According to what was discussed above, the distance decreases the temperature, increases the size of the clusters, and decreases the diffusion of the species, resulting in powder formation. Heterogeneous nucleation on the substrate promotes the growth of SiO*_x_* films. The distance between the filaments and the substrate is greater than 9 mm; this reduces the temperature below 700 K according to experimental and theoretical results, promoting the growth of films. Temperature control in these systems can be modulated through the longitudinal distance between filaments, sources, and substrates. The transverse distance between sources and filaments also plays an important role in the deposition process; however, the proposed 2D model limits the study of this effect.

### Velocity

**Figure 7:**
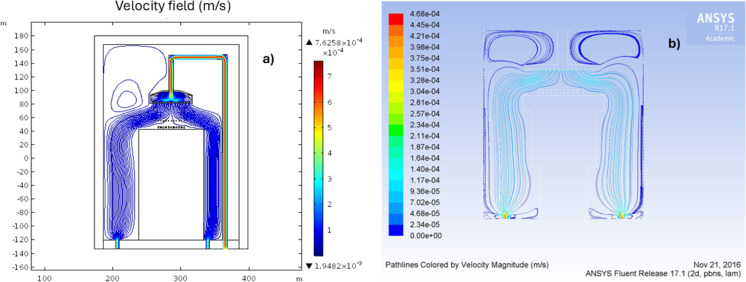
Velocity profile in the HFCVD reactor obtained with (a) COMSOL and (b) ANSYS.

Flow dynamics play a critical role when the deposition takes place at atmospheric pressure. Fluid velocity and distribution analysis were previously performed and analyzed by CFD using ANSYS Fluent [24], as depicted in Figure 7b. From this study, it was determined that it is necessary to make a change in the configuration of the reaction chamber outlets to homogenize the flow distribution and, thus, be able to optimize the deposition area to two inches. The previously reported ANSYS velocity profile is in very good agreement with that obtained in COMSOL, which is shown in Figure 7a. Both profiles in Figure 7 describe the laminar flow of the gas in zone 2 and the formation of a turbulent flow at the diffuser due to the interaction with the walls. In a study of the deposition of silicon dioxide using an atmospheric-pressure plasma-enhanced CVD reactor, the reactor performance was shown to be strongly affected by the flow dynamics [33–34].

### Distribution and concentration of species

**Figure 8:**
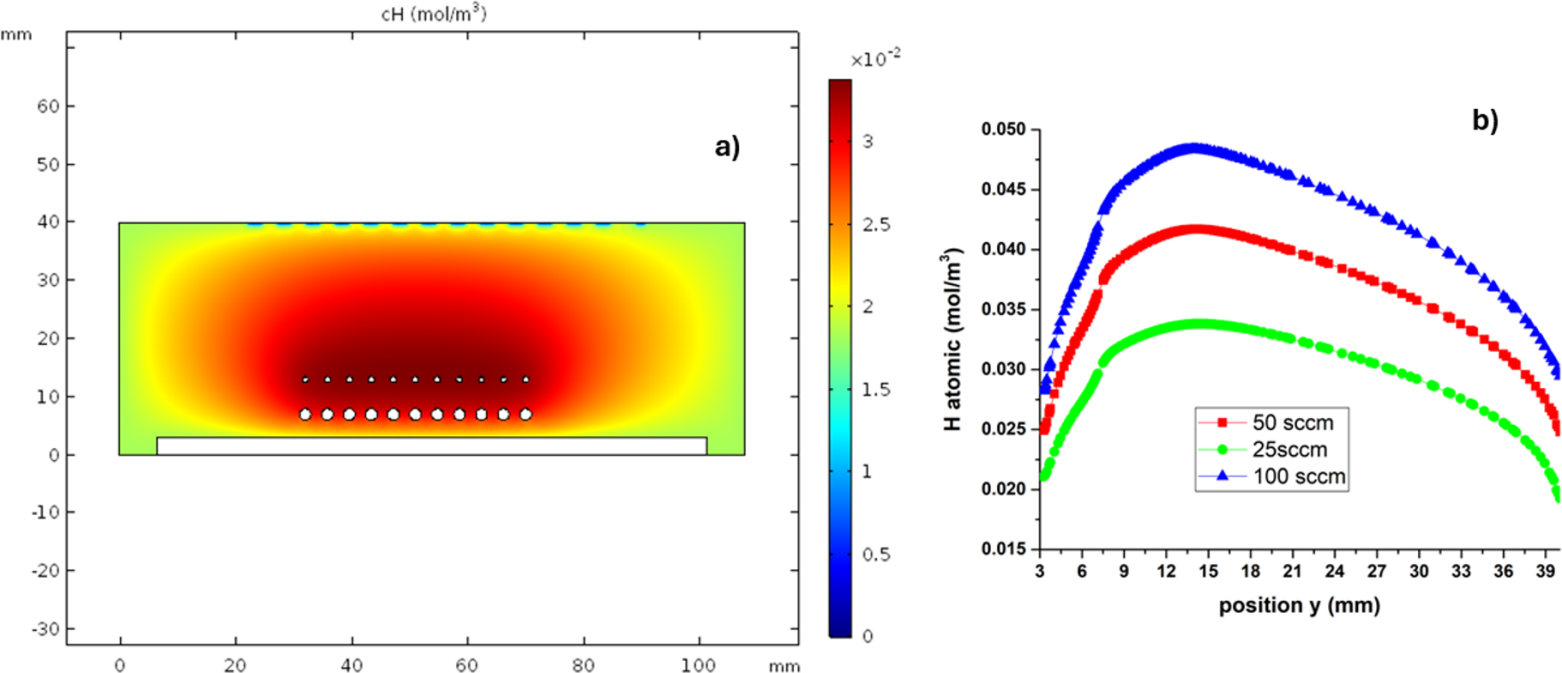
(a) Concentration map of atomic H in the deposition zone. (b) Concentration as a function of the position on the *y* axis.

As previously mentioned, temperature and fluid dynamics play a very important role in the formation and diffusion of precursor species. The solution of the model allowed us to obtain preliminary results for the concentration of the species in the reactions proposed in Table 3. The concentration profiles for H°, SiO, O_2_, and OH were analyzed at three different flow levels of 25, 50, and 100 sccm. The H° concentration map is shown in Figure 8a, and the concentration as a function of the *y* position is shown in Figure 8b. Figure 8b shows different concentration profiles for different H_2_ fluxes. The profiles vary slightly, and the concentration increases with the flux of H_2_. This effect was already studied experimentally, and the most significant results were the analysis of how the hydrogen flow influences the composition SiO*_x_* films by X-ray photoelectron spectroscopy; as the hydrogen flow increases, the concentration of Si also increases in comparison with that of oxygen, modifying stoichiometry and bandgap [35]. The distribution of H° in zone 2 is a result of the temperature distribution. The concentration is greater near the filaments, decreasing with distance along the *y* axis away from the quartz sources, where the temperature varies in the range of 800–1200 K. When the distances from the filament to the source are greater, no film deposition occurs. This effect is mainly due to the recombination of H° to H_2_ as the temperature decreases.

The formation of these gaseous species is an important step since they contain the radical species that give rise to the surface reactions for the growth of the films. In the reaction mechanisms reported for the growth of SiO_2_ films, the role of intermediate ^•^H and ^•^OH and ^•^O_2_ radicals has been pointed out through in situ Fourier-transform infrared spectroscopy. These species react with silane-derived and surface hydroxy groups, which leads to deposition. They are also responsible for the incorporation of Si–OH bonds into the silicon oxide matrix. The ^•^H and ^•^OH species attack unsaturated surface species with dangling bonds. Oxidation of surface hydrogens is the predominant reaction for O to convert H to OH. Finally, ^•^H and ^•^OH radicals can attack saturated surface species, removing H to form H_2_ and H_2_O, respectively, in the gas phase [21,36]. The presence of dangling bonds in the SiO*_x_* films was detected previously. In this study, SiO*_x_* films with thermal treatment and without thermal treatment were analyzed, and a decrease in thickness, refractive index, and excess silicon was observed. This behavior was attributed to the structural rearrangement at the atomic level by the thermal treatment due to the desorption of hydrogen from dangling bonds. The films after thermal treatment exhibited greater photoluminescence compared to those that did not undergo thermal treatment [29].

**Figure 9:**
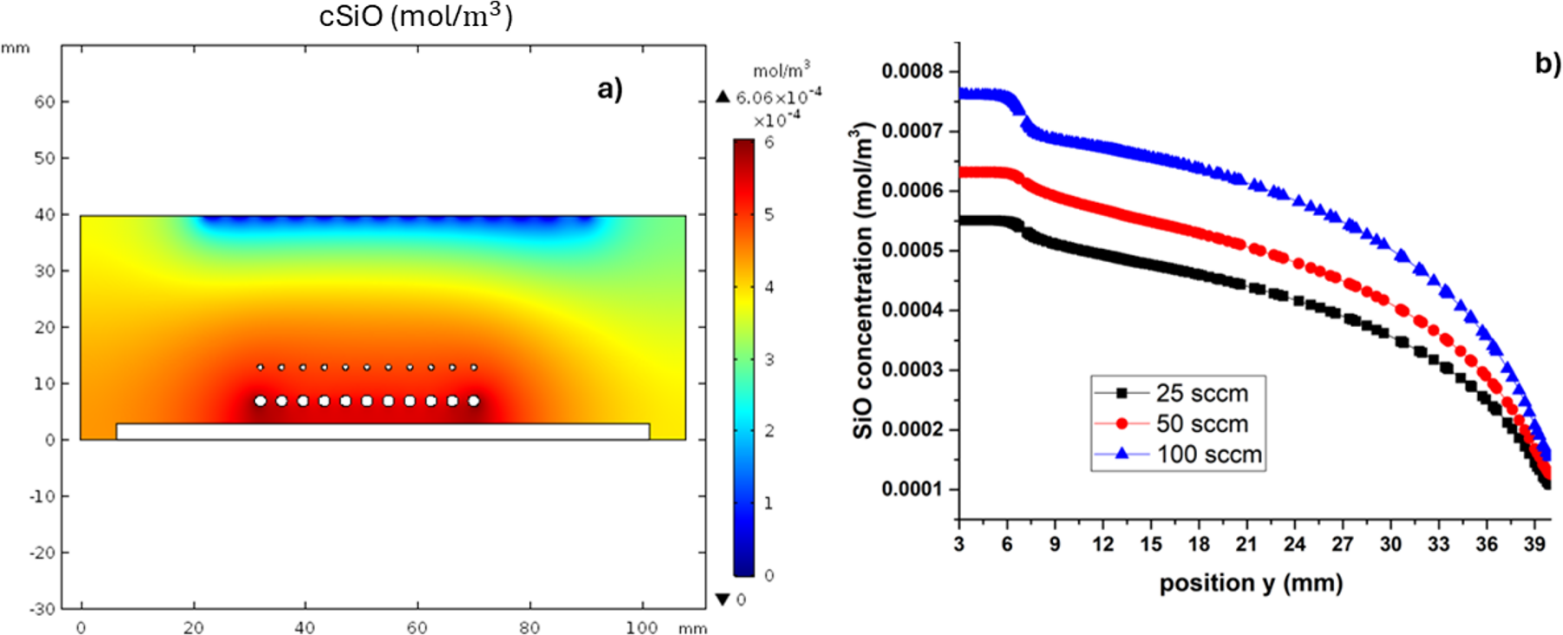
(a) Concentration map of SiO in the deposition zone. (b) SiO concentration vs *y* position.

The SiO species plays an important role in the proposed mechanism; the reaction of SiO with ^•^OH and ^•^H radicals leads to the formation of HSiO and silanes [15]. Silane and silanol species were also found in the equilibrium thermodynamic study; they can be the precursors of nanocrystalline silicon (nc-Si) in the SiO*_x_* films [24]. The nc-Si obtained by this technique has been studied and characterized [35]. The calculated concentration of SiO and OH is shown in Figure 9a. These species exhibit a higher concentration in the region among the quartz sources and in the vicinity of the substrate. These concentrations are generated in chemical equilibrium by reaction 3 in Table 3. Figure 9a shows the SiO concentration map, and Figure 9b shows the concentration as a function of the *y* position for different hydrogen fluxes.

**Figure 10:**
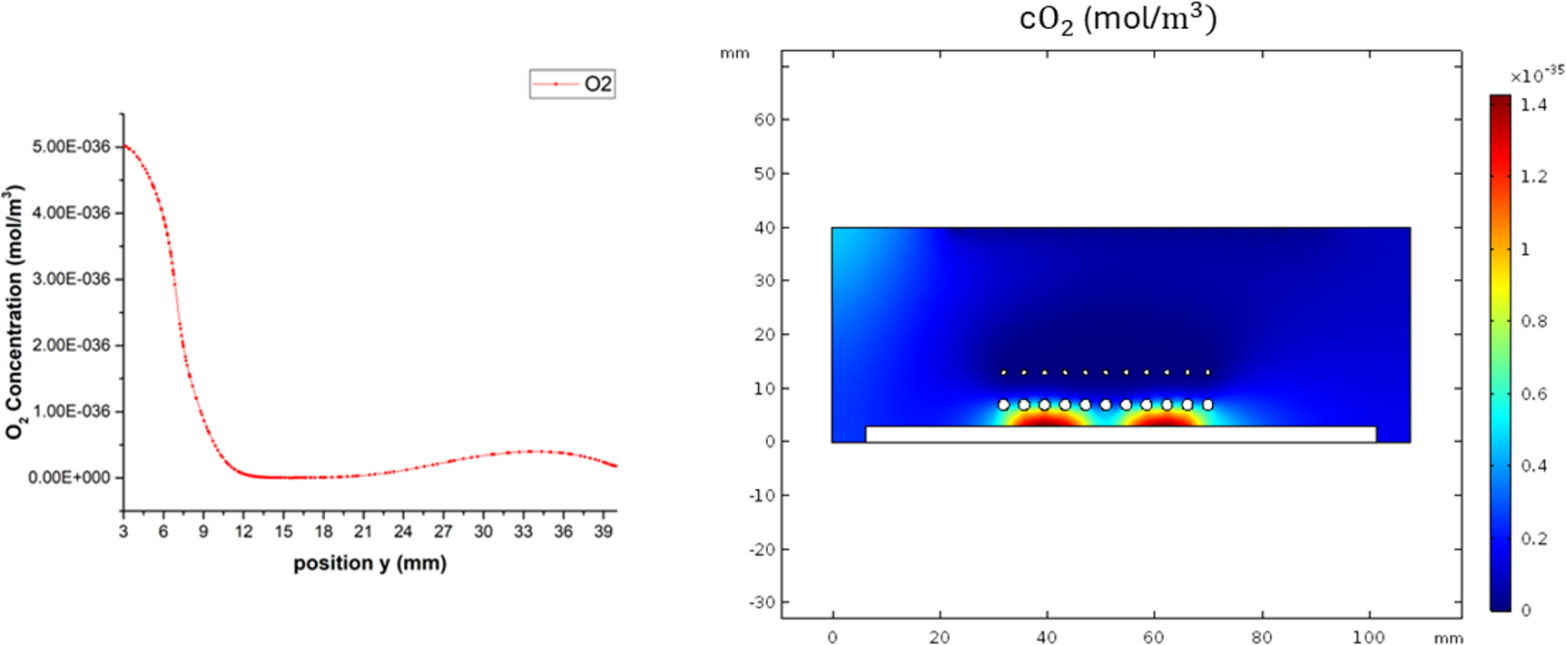
(a) Concentration of O_2_ as a function of the *y* position. (b) O_2_ concentration map in zone 2.

The oxygen in the deposition process is only from the solid quartz sources. In our model, there is not an additional source for O_2_. According to the proposed mechanism in reaction 4 in Table 3, the formed O_2_ reacts with H_2_ on the surface of the quartz sources to form OH. The O_2_ content is lower than that of the other species because it reacts with H_2_ and H°. Therefore, this species is predominantly found below the quartz sources. The O_2_ concentration map is shown in Figure 10b. The concentration of O_2_ above the sources is almost zero due to the reaction with atomic H leading to the formation of OH. Figure 10a shows the concentration of O_2_ as a function of the position.

## Conclusion

A model of the reaction mechanism of quartz sources with atomic hydrogen was established in thermodynamic equilibrium, and a thermochemical study of the heterogeneous chemical reaction was carried out using FactSage. We obtained the thermodynamic and transport properties to set up a 2D model. Through 2D simulation with COMSOL Multiphysics^®^ software, it was possible to obtain the concentration profiles of the main species H°, O_2_, OH, and SiO, which contribute to the growth of SRO films. In addition, the temperature and flow velocity profiles were obtained. Furthermore, it was possible to make a comparison between the theoretical results and those obtained from experiments. It was observed that the effect of the temperature on the distribution of the species is the most important. Regarding the temperature variation on the substrate, it was found that it is mainly caused by the increase in the distance between sources and substrate. The temperature on the substrate is influenced by radiation, conduction, und convection mechanisms. The transport of species is due to the convection and diffusion effects.

## Data Availability

Data generated and analyzed during this study is available from the corresponding author upon reasonable request.
